# Ambulant monitoring and web-accessible home-based exercise program during outpatient follow-up for resected lung cancer survivors: actual use and feasibility in clinical practice

**DOI:** 10.1007/s11764-017-0611-6

**Published:** 2017-04-10

**Authors:** J.G. Timmerman, M.G.H. Dekker-van Weering, M.M. Stuiver, W.G. Groen, M.W.J.M. Wouters, T.M. Tönis, H.J. Hermens, M.M.R. Vollenbroek-Hutten

**Affiliations:** 1 Roessingh Research and Development, Telemedicine Group, Enschede, The Netherlands; 2 Faculty of Electrical Engineering, Mathematics and Computer Science, Telemedicine Group, University of Twente, Enschede, The Netherlands; 3 The Netherlands Cancer Institute, Amsterdam, The Netherlands; 4 Ziekenhuis Groep Twente, Almelo, The Netherlands

**Keywords:** Lung cancer, Telehealthcare, Cancer survivorship, Rehabilitation, Feasibility, Implementation

## Abstract

**Purpose:**

The aim of this study is to evaluate the feasibility of a telehealthcare application for operable lung cancer (OLC) patients, consisting of ambulant symptom and physical activity monitoring (S&PAM) and a web-accessible home-based exercise program (WEP), and identify possible barriers for successful adoption and implementation.

**Methods:**

A two-stage mixed methods design was used, in which 22 OLC patients and their treating healthcare professionals (HCPs) participated from pre-surgery to three (stage 1; *n* = 10) or six (stage 2; *n* = 12) months post-surgery. Actual use and acceptability (usability, usefulness, and satisfaction) were evaluated.

**Results:**

Seventeen OLC patients (age (SD): 59 (8) years; 8 female) actively used the modules. S&PAM use varied from 1 to 11 monitoring days prior to outpatient consultations. Patients used WEP most frequently during the first 5 weeks, with an average of four logins a week. Fifty-eight percent used WEP beyond 7 weeks. No adverse situations occurred, and patients felt confident using the applications.

Perceived added value included active lifestyle promotion, decreased anxiety, and accessibility to specialized HCPs. Physiotherapists used WEP as intended. Contrarily, physicians scarcely used information from S&PAM. To promote future adoption, strategies should focus on high-level patient tailoring of the technology, and formalization of including the applications in the clinical workflow.

**Conclusions:**

Ambulant monitoring and web-accessible home exercise is clinically feasible for OLC patients. However, low level of adoption by referring physicians may hamper successful implementation.

**Implications for cancer survivors:**

Patients perceived both ambulant monitoring and web-accessible exercise as an added value to regular care and feasible to use in the period before and after lung resection.

**Electronic supplementary material:**

The online version of this article (doi:10.1007/s11764-017-0611-6) contains supplementary material, which is available to authorized users.

## Introduction

Lung cancer is associated with high symptom burden and high level of unmet needs during and after treatment [1]. Although lung resection has the best treatment outcomes in terms of survival in early stage lung cancer, resected patients are faced with an additional worsening of physical fitness, lung function, quality of life, and symptoms such as pain or fatigue following surgery [2–4]. Good quality survivorship care post-surgery is essential to optimize recovery and prevent rehospitalization. Physical training and ambulant symptom monitoring might promote recovery and optimize treatment outcome [5–8], yet availability and accessibility of such non-invasive interventions specifically adapted for operable lung cancer (OLC) patients remains extremely low [9].

Telehealthcare, defined as delivery of care by a healthcare professional over a distance using information and communication technologies (ICT) [10], is hypothesized to be a promising method to improve both the accessibility and quality of post-surgery cancer rehabilitation. Studies in various cancer diagnoses showed beneficial effects of telehealthcare on physical fitness, symptom management, and patient empowerment through frequent health monitoring, home-based exercise programs, and tailored information on disease and treatment [11–14]. Using the internet, smartphones, and sensors, telehealthcare services are accessible on patients’ demand, wherever and whenever they need, providing timely support and promoting health-related self-management behaviors. Currently, evidence is emerging that telehealthcare applications are also acceptable for OLC patients and clinically safe [13, 15, 8].

However, showing acceptability and clinical safety is not sufficient for successful adoption and widespread use in everyday care [16, 17]. The context of use is considered important as well [18, 17, 19], which means that insight in acceptability and use *within* this context is of utmost importance to make the potential of telehealthcare come true.

Therefore, the primary aim of this study was to evaluate the feasibility of a telehealthcare application when used in clinical practice. Research questions to be answered were: (1) how do patients and HCPs use the application during outpatient follow-up in terms of frequency and duration; and (2) is the application acceptable for patients and HCPs as offered. A secondary aim was to identify factors for successful adoption and implementation following the staged approach of Jansen-Kosterink and Vollenbroek-Hutten [16]. By doing so, essential factors can be detected in an early phase, which enables efficient modification of the application before larger-scale implementation.

## Methods

A repeated-measures, single-arm, mixed-methods feasibility study was performed from January 2014 to January 2016. The study was approved by the Institutional Review Board Netherlands Cancer Institute—Antoni van Leeuwenhoek Hospital (NL44192.031.13/N13POR), and all participants provided informed consent prior to participation in the study. The staged approach was used to guide the evaluation [16], resulting in a stage 1—optimization of usability for use in clinical practice—and a stage 2—evaluation of clinical feasibility—study.

### Sample and setting

Participants were recruited from the Netherlands Cancer Institute (NKI), Amsterdam, the Netherlands, between January–May 2014 (stage 1) and January–July 2015 (stage 2). Eligible participants were Dutch-speaking adults aged 18 years or older, diagnosed with primary non-small lung cancer (NSCLC) and scheduled for curative lung resection. Participants were identified during the multidisciplinary meeting at the NKI, and patients’ treating oncologist validated eligibility. A study information letter was sent to eligible patients, after which patients were contacted by the first author. When interested, a first appointment was scheduled at the NKI prior to surgery.

Participants were excluded if they had no access to a computer or internet, were unable to walk independently with or without walking aid (e.g., cane), exhibited severe cognitive disorders or emotional instability, or suffered from uncontrolled comorbidities. HCPs (oncologists, surgeons, physiotherapists) involved in the care of the included patient were also recruited.

### Intervention

The telehealthcare application, called the *Remote Monitoring and Treatment (RMT) application*, has been described previously [20]. Briefly, it consists of two modules: (1) a symptom and physical activity monitoring (S&PAM) system, and (2) a web-accessible exercise program (WEP) with remote supervision by a physiotherapist.

**Fig. 1:**
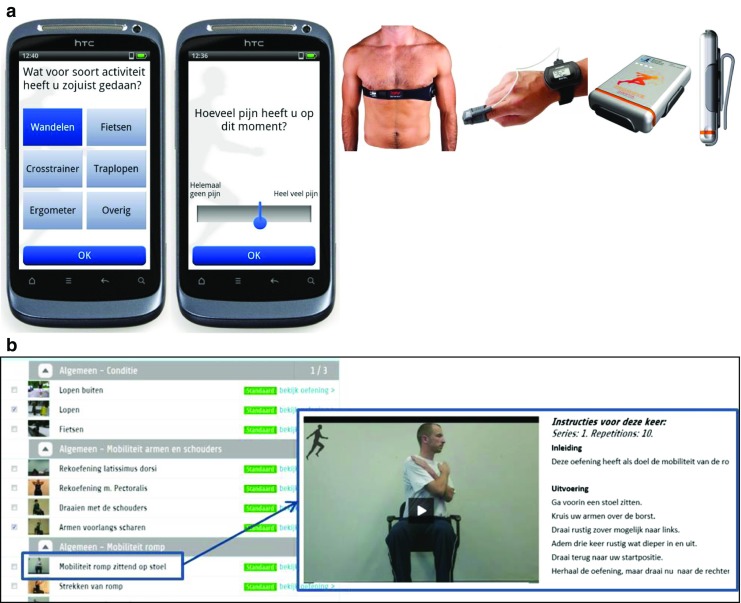
The remote monitoring and treatment (RMT) service for lung cancer patients. **a** The symptom and physical activity monitoring (S&PAM) system consisting of a smartphone, heart rate sensor, pulse oximeter, and accelerometer. **b** Example exercise of the web-accessible home-based exercise program (WEP) including a movie and written instructions as displayed in the online portal

*The S&PAM module* aims to increase insight in the severity of and change in self-reported symptoms, well-being, and daily physical activity. The system consists of an android smartphone—used as input device for self-rated symptom severity (pain, dyspnea, fatigue; scored by moving slider between 0 (‘no [symptom] at all’) to 10 (‘extremely/a lot of [symptom]’)), mood (*valence*, *calmness*, *energetic arousal*; scored from 0 (e.g., ‘tense’)—6 (e.g., ‘calm’) [21, 22]), weight, and pain medication use—and three on-body sensors, i.e., an accelerometer, heart rate sensor, and an oxygen saturation sensor (Fig. 1a). The symptom scores are combined with physiological parameters from the sensors and summarized into graphs, accessible for both patients and HCPs via a web portal, which is integrated with existing electronic medical records (EMRs) at the hospital.

*The WEP module* aims at improving physical fitness of the patient by means of an online, tailored exercise program, which is based on patients’ fitness and goals—assessed during a face-to-face intake—and is performed at home. Each exercise is illustrated by a movie with spoken instructions, and supported by written text (Fig. 1b). Progress is monitored via patient self-report (number of exercises successfully completed, and the experienced difficulty of the exercise) and automatically logged information on use (frequency, duration of login, number of page, and exercise views). If a patient experiences problems with exercise execution (e.g., unclear how to perform an exercise or non-acute bodily pain related to the exercise program), the patient can click a button on the web portal reading “cannot perform exercise, report to therapist”, which results in a standardized email sent to the responsible physiotherapist with instructions to contact the patient. For acute physical problems, the patient is instructed to contact his/her GP.

The modules were accessible for patients via ‘MyAVL’, the interactive patient portal from the NKI [12] but ran on the Continuous Care and Coaching Platform hosted at the research institute [19].

### Study procedures

#### Stage 1 study: preparation of the RMT application for clinical use

To optimize usability of the application for clinical use, the RMT application was offered to a small sample of operable NSCLC patients and their HCPs at the NKI from pre-surgery to 3 months post-surgery.

Patients, pulmonologists, and physiotherapists participated in telephone or face-to-face semi-structured interview to evaluate satisfaction 3 months post-surgery. All interviews were performed by the first author and lasted between 15 and 30 min. Participants were asked to describe how they used the application, what they thought of the ease of use, and if they had experienced any problems (e.g., technical) using the application. Their comments resulted in a list of critical requirements for adaptation of the RMT application. Results are summarized in this article and adaptations were realized before start of the stage 2 study.

#### Stage 2 study: evaluation of clinical feasibility of the RMT application in clinical care

Before start of the study, thoracic surgeons and pulmonologists of the NKI were given a 30-min presentation about content and possible benefits of the S&PAM module, where in the EMRs the data could be found and how these should be interpreted. Physiotherapists of the NKI were familiarized with the WEP portal during a 2-h workshop. Additionally, a paper manual was provided. Telephone and email support for the use of the modules was also available for both patients and HCPs.

#### Study protocol

**Fig. 2:**
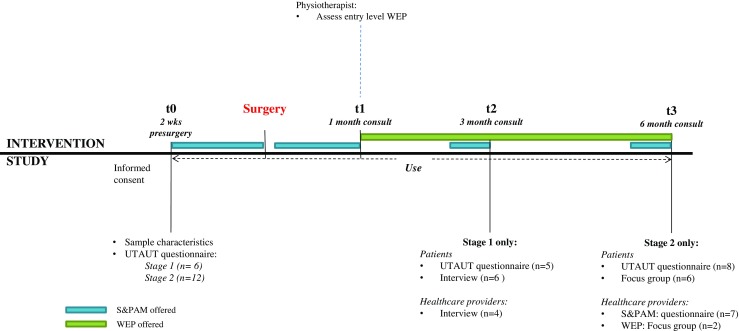
Study protocol stage 1 and stage 2 study. Stage 1 ran from 2 weeks prior surgery (t0) to 3 months post-surgery (t2) only. *S&PAM* symptom and physical activity monitoring module; *WEP* web-accessible home exercise program; *UTAUT* unified theory of acceptance and use of technology

The study protocol is summarized in Fig. 2.

##### Pre-surgery (t0)

At baseline, patients received the system for the S&PAM module, oral instructions, and a paper manual. Patients were asked to use the system in the period *before* surgery for a minimum of 3 days a week, preferably for 2 weeks. The first week following the appointment, the investigator checked if scores were displayed in the EMR, and when necessary, encouraged the patient to complete measurements before surgery.

##### Post-surgery

Outpatient appointments with the physician (thoracic surgeon or pulmonologists) and the physiotherapist were scheduled at 1 month post-surgery, as per usual care. After hospital discharge, the patients were contacted by the investigator and reminded to use the S&PAM system for a minimum of 3 days a week, preferably all weeks until the 1-month physician appointment at the hospital (t1).

##### First month post-surgery (t1)

Two days before the outpatient visit, physicians received an email with a reminder that additional information from the S&PAM system was available in the EMRs, including instructions where and how to access this information. Patients returned the S&PAM system during their visit to the hospital.

During the physiotherapist consultation, patients received user instruction for the WEP portal, including a personal login and a paper manual. A brief and individualized assessment of the patient’s fitness level was made, after clarifying individual exercise goals and preferences. A tailored exercise program was then constructed by the physiotherapist and made accessible to the patient within the first week following the appointment. The exercise program was adjusted at least once a month. If needed, the physiotherapist could adjust the program more often. No specific guidelines were given regarding the number or content of contacts between patients and the physiotherapists. Contact between patient and therapist was possible via chat messages on the portal. More complex issues, such as problems with performing exercises or changes in health, could be discussed by telephone or email. The WEP module was offered until 6 months post-surgery.

##### Three and 6 months post-surgery (t2 and t3)

Three weeks prior to the 3 and 6 months follow-up, the S&PAM system was again sent to the patients by the investigator, including written instructions for use. Patients were asked to use the system prior to their appointment for 3 days a week during 2 weeks. Two days before the appointment, physicians received a reminder that monitoring data was available for this patient in the EMR. Again, instructions were included where and how to access this information. Patients returned the S&PAM system during their follow-up visits to the hospital.

Dropout was defined as a patient who did not attend the final measurement at t3. Every patient was contacted to determine their reason(s) for dropping out.

### Data collection

#### Patient characteristics

At baseline, demographics such as sex, age, marital status, education level, and employment status were collected via a questionnaire. Experience with internet and computer technology was self-reported in terms of frequency and duration. Clinical information (diagnosis, cancer stage, treatment details, number of comorbidities, lung function) were obtained from the hospital medical records.

#### Actual use

##### Symptom and physical activity monitoring

To reflect use of the S&PAM module, the following measures were logged, extracted from the database, and further analyzed:

- *Patients:*
  - The number of days that patients used the monitoring system, calculated as the number of days that data were available from the system (accelerometer or symptom scores) expressed over all periods together as well as per treatment period (pre-surgery, first month following surgery, prior to 3 months consultation, and prior to 6 months consultation).
  - Frequency (number) and duration (minutes) of login on the S&PAM portal expressed over all periods together.

- *Physicians:*
  - Frequency (number) and duration (minutes) of login expressed over all weeks together.

##### Web-accessible home-based exercise program

For the WEP module, the following use measures were evaluated:

- *Patients:*
  - Number of weeks patients used the service, measured as number of weeks from the first to the last week that a patient logged in on the portal
  - Frequency (number) of logins per week
  - Average duration (minutes) of login per session
  - Reason for ending service (provided by patients through self-reported)
- *Physiotherapists:*
  - The frequency (number) and duration (minutes) of login expressed over all weeks together were logged.
  - Time needed to perform the first consultation (including instruction), setting up and adapting a tailored exercise program, as recorded by the therapist per patient.

## Acceptability

A combination of quantitative and qualitative measures was used to measure acceptability of the S&PAM and WEP modules in both patients and HCPs.

### Patients

An *online questionnaire* based on the Unified Theory of Acceptance and Use of Technology (UTAUT) [23] was administered at pre- (t0) and post-intervention (t3). The modules (S&PAM and WEP) were evaluated independent from each other in the questionnaire. Scores prior to surgery give an indication about the *expectations* patients had about the RMT service, while the scores at study completion represent their *experiences* or *acceptability* of the RMT service. The questionnaire evaluated the components *effort expectancy* or usability, *performance expectancy* or usefulness, *social influence*, *behavioral intention to use*. Facilitation conditions were measured in terms of *perceived self-efficacy*. Computer availability and internet access were inclusion criteria for participation and therefore not measured as part of facilitation conditions. *Attitude* and *satisfaction* were added, as they are hypothesized to influence intention to use and actual use [24, 18, 25]. Each item was phrased as a statement, and scored on a 7-point Likert scale (from completely disagree to completely agree). Negative phrased items were transformed so that a higher score indicated higher expectation/experience. For each component, the item scores were summed, and the average score was taken as the final outcome measure.

At the end of the study, *a patient focus group* was performed by the investigator. Discussion focused on usability and perceived usefulness of the application for resected lung cancer patients, patients’ motivation for use, and if they thought the application should be part of standard care for resected lung cancer patients (intention to use). The first aim was to generate input for improvement of the application itself. The secondary aims were to gather information about the clinical value of the application as well as influencing factors that motivate or hamper patients to use these kinds of applications. The focus group was recorded with permission of the participants and notes were taken.

### Healthcare professionals (HCPs)

Acceptability of the S&PAM module was evaluated via an online questionnaire at the end of the study (January 2016). The questionnaire was send to 16 HCPs that are involved in the outpatient care of operable lung cancer patients (two specialized oncology nurses, four thoracic surgeons, ten pulmonologists).

If an HCP indicated that he/she had not used the results of the S&PAM module, reasons for non-use were registered as well as the expected usefulness of symptom monitoring in clinical care for lung cancer patients.

A focus group with physiotherapists who used the WEP module was performed by the first author at the end of the study to evaluate acceptability of the WEP module in terms of usability, usefulness, satisfaction, and intention to keep using the module.

### Data analysis

IBM’s Statistical Package for the Social Sciences (SPSS, 23) was used for the statistical analyses. Demographic, patient expectation and experience (UTAUT components) were calculated as frequencies (percentage), medians, and interquartile ranges.

For evaluating the actual use of the web portals of S&PAM and WEP all logins less than 2 min in duration were excluded from analysis. Following, logins that occurred on the same day were considered a single session and duration of these sessions were summed. Means, standard deviations, and ranges were calculated of the use measures. For visualization of actual use, dot plots were generated capturing each individual use values, means, and 95% confidence intervals (95% CI). CI’s were calculated using the *t*-distribution due to small sample size.

For qualitative insight into feasibility and acceptability, notes of the focus groups were combined with results of the questionnaire to highlight the most important aspects. Responses on the HCPs questionnaire were summarized using descriptive statistics.

## Results

### Stage 1 study

The RMT service was offered to 10 patients (60% female; median age (IQR): 56.6 (52.8–62.8) years), of which eight patients used one or both modules of the RMT service. Seven physicians were involved in the care of these patients (three surgeons, four pulmonologists). Detailed description of patient characteristics can be found in Online Resource 1. In post-intervention, patients indicated good usability and usefulness, confidence in using the modules (‘self-efficacy’), and a positive attitude and intention to use the modules (i.e., all components a median score of >5 out of 7). Overall, patients felt satisfied with the modules, rating the S&PAM and WEP modules with a 5.3 (4.5–6.5) and a 5.6 (4.5–6.9), respectively (out of 7). Four patients (two female) and four HCPs (two physiotherapists, two pulmonologists) participated in the semi-structured interviews. Two patients declined participation in a personal interview, but gave written comments on the questions from the interview. From the interviews and written comments, eight critical issues were extracted for the S&PAM and five critical issues for the WEP module. Critical issues and the functional requirements for adaptations have been summarized in Online Resource 2.

### Stage 2 study

#### Sample

Eighteen NSCLC patients scheduled for curative lung resection were approached for participation. Twelve patients agreed to participate (33% female; median age (IQR): 59.5 (54.5–66.0) years); a consent rate of 67%. Detailed description of patient characteristics can be found in the Online Resource 1. Most important reasons for refusal were surgery before enrollment could take place (*n* = 2), too little experience with computers/internet (*n* = 2), or emotional burden (*n* = 1). Following enrollment, four patients dropped out of the study, three prior to intervention, and one after 2 months of participation. Reasons for dropout were cancelation of the surgery (*n* = 2), emotional burden (*n* = 1), or complications following surgery (*n* = 1). Internet experience was high in this sample, with all patients indicating using the internet almost every day for more than 3 years. Twelve physicians were involved in the care of enrolled patients (four surgeons, eight pulmonologists).

#### Actual use of the modules

## Ambulant symptom and physical activity monitoring (S&PAM)

### Ambulant S&PAM system

**Fig. 3:**
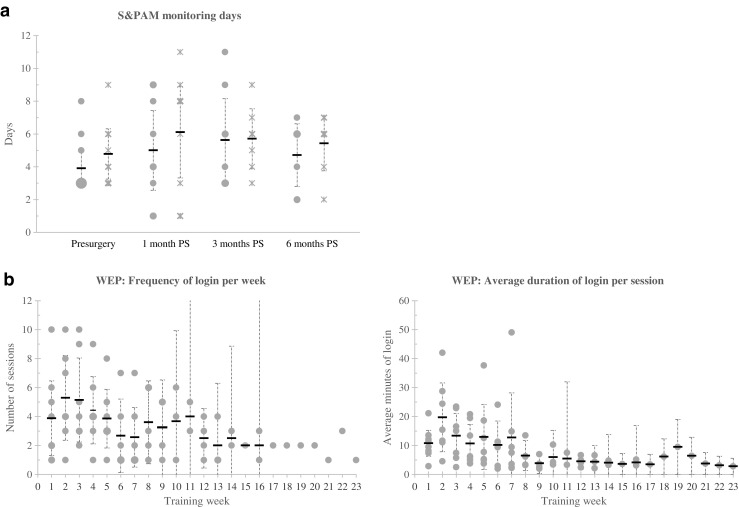
Use of the RMT application. **a** S&PAM: number of days with physical activity data (*dots*) and symptom scores (*crosses*); *PS* post-surgery. WEP: **b** frequency of login on the WEP portal per training week; **c** average duration of login per session per training week. *Training week 1* indicates the first week that a patient logged in on the web portal. A patient data point is represented by a *dot/cross*. Data points that overlap each other are visualized with *larger dots/cross*; the more overlapping points the *larger the dot/cross*. The average (*horizontal lines*) and 95% CI (*vertical lines*; calculated with *t*-distribution) are given

All patients used the S&PAM system at least once, resulting in 179 monitoring days. On average, patients used the system between 5 to 6 days per treatment period (i.e., pre-surgery, 1, 3, and 6 months post-surgery (Fig. 3a).

During the study, in three patients, heartrate and oxygen saturation monitoring was removed from the system. One patient experienced problems with attachment of the heartrate sensor because of his thorax wound post-surgery. One patient was anxious about using these sensors and for the other patient there simply was no sensor available. Technical issues were reported by six patients, but all issues could be resolved remotely. Most often reported was a loss of connection between the pulse oximeter and the smartphone during increased physical activity (*n* = 4). Two patients indicated problems rating the symptoms on the smartphone prior to surgery (t0). As can be seen from Fig. 3c, there are in general fewer days with PA data than there are for subjective symptoms. This was caused by low quality data in terms of missing data points.

### Web portal

In total, patients logged in 28 times on the portal to view the results of the symptom monitoring. Nine patients logged in at least once. Mean duration of login was 8 min (min-max: 2–35 min). On average, patients clicked on 11 different pages per session (SD = 12 page clicks). Patients looked most to the pages containing the detailed information about heart rate and oxygen saturation (40% of all page clicks), followed by the summary of symptom scores and daily activity (33% of all page clicks).

Of the 12 physicians involved, only three physicians logged in on the portal (once each), with an average login time of 12 min.

## Web-accessible home-based exercise program (WEP)

Eight patients (67%) used the exercise portal at least 1 week following lung resection. Patients started 4 (*n* = 3), 5 (*n* = 2), 6 (*n* = 2), or 7 (*n* = 1) weeks following resection. Use of the portal in terms frequency of use per week and average duration per session are visualized in Fig. 3b/c. Patients used the exercise portal most frequently in their first 5 weeks of use. Seven patients (58%) used the portal for 7 weeks or longer. During the program, none of the patients used the shortcut button to indicate difficulty in exercise performance or reported an acute, serious problem as a result of the exercise program. Twenty percent of all sessions lasted 20 min or longer; while the majority of all sessions (66%) lasted less than 10 min.

Half of the patients reported that after some weeks practicing at home, they printed the exercise program and performed the exercises at a local fitness center (*n* = 3) or community center (*n* = 1). Reasons for exercising in these centers were availability of better equipment (*n* = 1), support from trainer (*n* = 1), and ‘used to go to the fitness center prior to surgery’(*n* = 2). Reasons for ending were that patients felt that they had reached their fitness goals and were fit enough to pick up their usual exercise activities (*n* = 5), outpatient follow-up ended (*n* = 1), or that exercises were too easy (*n* = 2).

Two physiotherapists participated in the study. They logged in 46 times during the study, with a total duration of 805 min. Time investment per patient was as follows (mean ± SD): intake with the patients 60 ± 7 min (including 30 min consultation part of ‘standard’ care), first set-up of exercise program 35 ± 9 min, and adaptation of the training program 19 ± 7 min.

## Acceptability

Eight patients completed the online questionnaire at both t0 and t3, and six of these patients participated in the patient focus group. Dot plots of the results from the online patient questionnaire can be found in Online Resource 3. Seven HCPs completed the online questionnaire; five pulmonologists and two specialized oncology nurses.

### Ambulant symptom and physical activity monitoring (S&PAM)

#### Patients

At t0, patient expectations were generally high (medians >5 out of a score of 7), with the exception of usability (median (IQR): 4.3(4.0–6.0)), and all patients had a positive intention to use the S&PAM module.

Following the intervention (t3), most patients indicated that the monitoring system had good usability and all felt competent using the module (that is, score >5 on perceived self-efficacy). Patients felt satisfied with the module and had the opinion that the module should be offered by the hospital to all eligible OLC patients as part of standard care (median satisfaction score =6.0 (IQR = 5.6–6.2); median intention to use score =6.0 (IQR = 5.0–6.8), respectively). On average, scores for usefulness indicated that patients experienced benefit using the system during treatment. Nevertheless, three patients scored rather low on usefulness (score between 3 and 4). Qualitative data show that they were disappointed with the lack of feedback from their physician on the results of the S&PAM, which made the module less useful, and, as a consequence, decreased motivation to use the S&PAM system. During the focus group, this decline in motivation was confirmed by all other patients. As a result, patients felt no need to extend measurements beyond the prescribed frequency. Next to that, patients also requested tailoring of the monitoring protocol in terms of monitoring frequency and sensors employed, based on their individual pattern of recovery and needs.

Patients mentioned several points of improvements regarding usability. Connection problems between pulse oximeter sensor and smartphone during activity (*n* = 4), and difficulty understanding visualization of the results on the portal (*n* = 4) were the aspects mentioned most often.

#### Healthcare professionals

The majority of the HCPs (*n* = 5) expected added value of a symptom monitoring system in the care for lung cancer patients in that it might improve insight into the capacity, symptoms, and daily activity, and with this information, improve treatment choices. Most HCPs reported that they would use the S&PAM during chemo (*n* = 4). But in their opinion, also surgery (*n* = 3), concurrent (*n* = 2), and radiation therapy (*n* = 2) might benefit from the use of the S&PAM module.

Yet, only two pulmonologists who completed the online questionnaire indicated to be aware that their patients had used the S&PAM module. None of these HCPs actually checked the results of the monitoring in the EMRs. Reasons mentioned for not using the information from S&PAM were unawareness that a specific patient participated in the study, lack of time to check the information, and non-compatibility with the content and process of their work.

From a HCP perspective, most relevant to optimize future use was to emphasize and improve HCPs’ perception of the added value of the symptom monitoring system (*n* = 7).

## Web-accessible home-based exercise program (WEP)

### Patients

Prior to intervention (t0), expectation scores were high with all UTAUT components median scores higher than 5, and all patients had the intention to use the WEP module as much as needed.

In the following use, most patients were satisfied with usability of the module, except for two (score <5) since the program was difficult to access on mobile phone, which hampered execution of the program on a different location than home. Nonetheless, all patients felt confident in their ability to use the module (“self-efficacy”).

Seven patients found the WEP module useful (score >5 out of 7), but one patient scored extremely low (2 out of 7). During the focus group, this patient indicated a lack of interaction with the physiotherapist, insufficient tailoring of the exercises, and lack of insight in progression as most important reasons for dissatisfaction. The majority of patients (*n* = 7) had the opinion that the module should be accessible to all operable lung cancer patients (score ≥5 on intention to use).

### Physiotherapists

Overall, physiotherapists indicated satisfaction and voiced a positive intention to keep using the WEP module. Therapists found it easy to provide user instruction to the patient, due to the simplicity of the patient portal. However, some suggestions for improvement were given for the therapist portal, including navigation between the summary of patient reported progress and corresponding exercises (to quickly adapt instructions based on the progress), and rearrangement of visualization of the chats (i.e., all the chats into one ‘chat-roll’ with a clear mark for messages that have been read).

Regarding usefulness, therapists believed that the module might contribute to improved accessibility of a cancer rehabilitation program and support of patients towards an active lifestyle by decreasing anxiety for physical activity. For improvement, a smartphone-supported application was considered likely to improve accessibility even more. In general, the necessary time investments were regarded acceptable to the therapists. It appeared critical to instruct the patient when and for what to use the chat function of the module as to prevent unmanageable number of messages.

To ensure successful implementation of the module, therapists advised a more blended care approach, that is, a combination of supervised and home-based training as to facilitate adequate evaluation of patients’ fitness and optimize program tailoring. Also, official (organizational) agreements are needed to ensure financing of the treatment and time to use the module next to face-to-face patientcare.

## Discussion

This study aimed to evaluate the clinical feasibility in terms of actual use and acceptability of a telehealthcare application, the Remote Monitoring and Treatment (RMT) application, for lung cancer patients treated with lung resection when used in daily clinical practice. Our findings suggest that the use of remote monitoring and treatment is feasible to lung cancer patients when offered pre- and post-surgery. Patients actively used the modules prior and following surgery, and perceived both modules as a beneficial contribution to their care. Also, the continuous treatment and involvement of ‘experts’ from the cancer institute, who were expected to be better informed about cancer and the treatment patients had received, was seen as a big advantage of using ICT-supported rehabilitation. These results are in line with previous studies that showed willingness of lung cancer patients to exercise at home following surgery [15] or daily rate symptoms on a smartphone during radiotherapy [11].

During the study, several issues emerged that need consideration for successful patient adoption. One of the important factors observed is usability of the RMT application. Results show that participants felt confident using on-body sensors, a smartphone, and corresponding protocols. This positive attitude might have been facilitated by a perceived usefulness of the system as well as a flexible monitoring protocol (e.g., change to a different monitoring protocol that did not include heart rate and pulse oximeter sensor) and availability of (paper) manual and a ‘helpdesk’. These factors have previously been linked to the acceptance of personal health devices in patients with chronic conditions [26]. Unfortunately, the S&PAM module suffered from loss of connection between the pulse oximeter and the smartphone during performing physical activities. Patients felt annoyed by this problem and it resulted in loss of data for the HCPs. Although SpO_2_ is considered clinically relevant both pre- and post-surgery for lung cancer patients [27, 28], it should only be included in the monitoring protocol when the sensor’s performance is adequate and reliable in the ambulant setting. Also, previous studies have shown the added value of S&PAM without SpO_2_ measurement [11, 8]. Therefore, we recommend that only patients at risk for desaturation should be offered the pulse oximeter [28].

For the WEP, the main usability issue reported was accessibility of the program from a mobile platform such as a smartphone. Providing the program on smartphone or tablet may facilitate patient use, due to accessibility at various locations which is preferred by patients (e.g., fitness center, local therapist, or communal centers), but also accessibility by broader (e.g., elderly) population due to increasing number of people possessing tablets rather than desktops [29].

Another factor that influences patient use is motivation. For both modules, patients explicitly reported a perceived decline in motivation towards the end of the study, caused by a lack of sufficient feedback by physicians and the system, and insufficient tailoring of the modules to the needs and capacity of the patients. In agreement, Hoaas et al. recently reported that providing patients with (objective) signs of improvements as well as treatment tailoring in terms of individual goal setting were seen as most important for COPD patients maintaining motivation to participate in a long-term telerehabilitation program [18]. Lack of motivation is not unique to the use of telehealthcare applications, but has been the object of evaluation in other, not ICT-related, interventions that focus on behavior change, such as promoting physical exercise [30]. Tailoring, or personalization, of treatment, and feedback is considered beneficial for treatment compliance and long-term behavior change [31, 32]. Evidence is growing that shows the potential and unique capability of technology to provide high level of personalization through monitor on an individual level and translate this complex gathering of information into tailored feedback and treatment [33–35]. Adding a gaming layer (‘serious gaming’), a virtual coach, or social component such as online group-based exercise to the program, may further enhance motivation [35]. Given the fast technological developments, we should strive for an individual, holistic approach that takes into account the complex interaction between the patient—including his/her health, norms, beliefs, and goals—the context and changes herein. For our RMT application, this means that we should utilize information about the patient (such as age, sex, cancer stage, experience with technology), his/her treatment (including treatment phase, care processes) to tailor the intervention protocol, and adapt the protocol frequently based on newly acquired information. Next to tailoring, other motivational strategies have been reported by our patients and HCPs that might improve the use and adherence of the RMT service. The most promising improvements were providing feedback about health and recovery via a smartphone app instead of a web-based portal since it is readily available and more user-friendly; and a more blended treatment approach, that is combining face-to-face consultation with home-based treatment as to optimize continuous tuning of the treatment to the patient.

In contrast with the overall positive findings regarding feasibility and acceptability from patients, the findings from HCPs were mixed. From the HCPs’ perspective, both modules were regarded beneficial and a valuable addition to care for operable lung cancer patients. However, evaluation of HCP satisfaction with the S&PAM module was unsuccessful, since physicians that completed the questionnaire all indicated non-use of the module. Since HCPs play a key role in making innovations available to patients and the influence they may have on the adoption and adherence of patients [20, 17, 36], active involvement of HCPs is regarded crucial for successful implementation of telehealthcare and should be given more attention. In line with a recent publication of Vollenbroek et al. [17], our results also show that a process of co-creation of the telehealthcare applications and protocols that are considered useful for their patients, it was clearly not enough for physicians to actually start using these modules as part of their daily practice. Interestingly, in our study, large variation was observed in HCPs using the modules. Physiotherapists that participated in the study showed high level of involvement, while physicians hardly used the information from the S&PAM module in their patients’ consultation. Research suggests that ease of use, perceived usefulness, and organizational- and work-related factors play an important role in HCP adoption [37, 19]. In our current study, it is difficult to draw firm conclusions about facilitating and impeding factors due to the small sample size. However, since ease of use and perceived usefulness of symptom monitoring were rated positive in our current and previous study by HCPs [20], the low use in clinical practice suggest a role of these work- and organization-related factors. For example, for the WEP module, the physiotherapists explicitly reserved time in their schedule for usage of the module, and they were solely responsible for the content of the exercise program and also served as the primary contact for patients regarding the WEP module. In contrast, no explicit agreements were made with physicians in terms of their role and responsibilities, and no time was reserved for learning and working with the S&PAM. Another issue might be that only few patients used the S&PAM, causing that usage of the monitoring data was not part of their daily routine. Lastly, the intervention protocol lacked clear guidelines how the modules and the results generated, should be integrated with hospital-based care. For example, specific cut-off scores for symptom levels were lacking, as was a clear protocol for handling alarming results of the S&PAM such as high pain scores.

This study provides valuable insight in how adoption and implementation of the RMT application in clinical practice can be promoted. While the technology can be further improved, we believe that integration of the RMT application with existing care processes is of paramount importance. This is in line with the work of Jansen-Kosterink, who states that it is not the technology, but the way the technology is embedded in care processes (‘service configuration’) that defines the service [38]. Therefore, implementation strategies should focus on formalization of the tasks and processes needed for usage of telehealthcare in practice, which should include the availability of time, funding and creating leadership [37, 19], and education of all involved personnel about the benefits of the service, as well as defining clinical protocols for the resulting information. Additionally, a stage 3 (large-scale) evaluation study is needed to evaluate effectiveness (clinical outcomes), adoption, adherence, and cost-effectiveness when used in clinical practice [16].

## Strengths and limitations

This study is one of the few that evaluated feasibility of ambulant symptom monitoring and web-accessible home-based exercise in clinical practice for lung cancer patients that underwent lung resection. We used the staged approach, resulting in a two-stage study, that allows for gradual development and fine-tuning between technology and clinical context. Several limitations of our study need consideration. First, our convenience sample might have resulted in inclusion of ‘enthusiasts’, resulting in higher IT literacy and levels of use. Also, patients with complications or who experience high levels of stress as a result of diagnosis are less likely to participate in the study. Exclusion due to lack of experience with or access to IT is a well-known barrier for successful adoption and implementation of telehealthcare in the elderly [39]. Currently, there are no reliable data available on IT literacy for this specific subgroup of patients, but the low percentage (i.e., 11%) of patients excluded in the current study for this reason probably is a too optimistic figure for the population as a whole. On the other hand, the number of chronically ill and elderly that have access to the internet and own a smartphone or tablet is increasing fast in the Netherlands [29, 40]. For example, in 2016, 82% of chronically ill patients were estimated to have internet access [40], and 63% of people 65+ years used a smartphone [41]. These numbers suggest that a lack of IT literacy as a barrier for participation will decrease in the upcoming years for elderly and chronically ill patients.

Second, the first author performed several research activities, including recruitment, S&PAM instruction, and leading the patient focus group. Therefore, patients might have been less willing to report negative comments during the focus group. However, the questionnaires were completed anonymously and resulted in better rating of the modules than the results during the focus group, suggesting that this effect can largely be ignored. Lastly, in the present study, we did not report effect of the intervention on treatment outcome and patients’ health or costs of the intervention. Although patients experienced the intervention as a beneficial addition to their treatment, additional research is needed to confirm clinical effect and cost-effectiveness objectively and in a larger sample.

## Conclusion

Our study demonstrates that remote monitoring and treatment is feasible to lung cancer patients when offered pre- and post-surgery. Patients actively used the ambulant symptom monitoring and web-based exercise modules prior and following surgery, and perceived both the modules as a beneficial contribution to their care. However, we also showed that a low level of adoption by referring physicians may hamper successful implementation.

A stage 3 (large-scale) evaluation study is needed, with focus on both involvement of HCPs through organizational formalization and adequate education, as well as promotion of patient motivation through individual tailoring of treatment content and feedback. Following the staged approach, outcome should evaluate clinical effect (in relation to the goal of each module), costs (in time and money), and factors that determine use and effect in both HCPs and patients.

## Electronic supplementary material

Online Resource 1 (DOCX 21 kb)

Online Resource 2 (DOCX 22 kb)

Online Resource 3 (DOCX 57 kb)

## References

[1] Maguire R, Papadopoulou C, Kotronoulas G, Simpson MF, McPhelim J, Irvine L (2013). A systematic review of supportive care needs of people living with lung cancer. Eur J Oncol Nurs.

[2] Brunelli A, Xiume F, Refai M, Salati M, Marasco R, Sciarra V (2007). Evaluation of expiratory volume, diffusion capacity, and exercise tolerance following major lung resection: a prospective follow-up analysis. Chest.

[3] Brunelli A, Socci L, Refai M, Salati M, Xiume F, Sabbatini A (2007). Quality of life before and after major lung resection for lung cancer: a prospective follow-up analysis. Ann Thorac Surg.

[4] Sarna L, Cooley ME, Brown JK, Chernecky C, Elashoff D, Kotlerman J (2008). Symptom severity 1 to 4 months after thoracotomy for lung cancer. Am J Crit Care.

[5] Ni HJ, Pudasaini B, Yuan XT, Li HF, Shi L, Yuan P. Exercise Training for Patients Pre- and Postsurgically Treated for Non-Small Cell Lung Cancer: A Systematic Review and Meta-analysis. Integr Cancer Ther. 2016**.** 10.1177/1534735416645180 PMC5736064 27151583

[6] Cavalheri V, Tahirah F, Nonoyama M, Jenkins S, Hill K (2014). Exercise training for people following lung resection for non-small cell lung cancer—a Cochrane systematic review. Cancer Treat Rev.

[7] Crandall K, Maguire R, Campbell A, Kearney N (2014). Exercise intervention for patients surgically treated for Non-Small Cell Lung Cancer (NSCLC): a systematic review. Surg Oncol.

[8] Cleeland CS, Wang XS, Shi Q, Mendoza TR, Wright SL, Berry MD (2011). Automated symptom alerts reduce postoperative symptom severity after cancer surgery: a randomized controlled clinical trial. J Clin Oncol.

[9] Rueda JR, Sola I, Pascual A, Subirana CM (2011). Non-invasive interventions for improving well-being and quality of life in patients with lung cancer. Cochrane Database Syst Rev.

[10] McLean S, Protti D, Sheikh A (2011). Telehealthcare for long term conditions. BMJ.

[11] Maguire R, Ream E, Richardson A, Connaghan J, Johnston B, Kotronoulas G (2015). Development of a novel remote patient monitoring system: the advanced symptom management system for radiotherapy to improve the symptom experience of patients with lung cancer receiving radiotherapy. Cancer Nurs.

[12] Kuijpers W, Groen WG, Oldenburg HS, Wouters MW, Aaronson NK, van Harten WH (2015). Development of MijnAVL, an interactive portal to empower breast and lung cancer survivors: an iterative**,** Multi-Stakeholder Approach. JMIR Res Protoc.

[13] Dickinson R, Hall S, Sinclair JE, Bond C, Murchie P (2014). Using technology to deliver cancer follow-up: a systematic review. BMC Cancer.

[14] Groen WG, Kuijpers W, Oldenburg HS, Wouters MW, Aaronson NK, van Harten WH (2015). Empowerment of cancer survivors through information technology: an integrative review. J Med Internet Res.

[15] Hoffman AJ, Brintnall RA, Brown JK, von Eye A, Jones LW, Alderink G (2014). Virtual reality bringing a new reality to postthoracotomy lung cancer patients via a home-based exercise intervention targeting fatigue while undergoing adjuvant treatment. Cancer Nurs.

[16] Jansen-Kosterink S**,** Vollenbroek-Hutten M. A Renewed Framework for the Evaluation of Telemedicine. 8th International Conference on eHealth, Telemedicine, and Social Medicine: eTELEMED 2016; April 24–28, 2016; Venice, Italy 2016.

[17] Vollenbroek-Hutten M**,** Tabak M**,** Jansen-Kosterink S**,** Dekker M, editors. From telemedicine technology to telemedicine services. Proceedings of the 3rd 2015 Workshop on ICTs for improving Patients Rehabilitation Research Techniques; 2015: ACM.

[18] Hoaas H, Andreassen HK, Lien LA, Hjalmarsen A, Zanaboni P (2016). Adherence and factors affecting satisfaction in long-term telerehabilitation for patients with chronic obstructive pulmonary disease: a mixed methods study. BMC Med Inform Decis Mak.

[19] Judi HM, Razak A, Sha’ari N, Mohamed H (2009). Feasibility and critical success factors in implementing telemedicine. Inf Technol J.

[20] Timmerman JG, Tönis TM, Dekker-van Weering MGH, Stuiver MM, Wouters MWJM, van Harten WH (2016). Co-creation of an ICT-supported cancer rehabilitation application for resected lung cancer survivors: design and evaluation. BMC Health Serv Res.

[21] Kanning M (2012). Using objective, real-time measures to investigate the effect of actual physical activity on affective states in everyday life differentiating the contexts of working and leisure time in a sample with students. Front Psychol.

[22] Wilhelm P, Schoebi D (2007). Assessing mood in daily life: structural validity, sensitivity to change, and reliability of a short-scale to measure three basic dimensions of mood. Eur J Psychol Assess.

[23] Venkatesh V, Morris MG, Gordon BD, Davis FD (2003). User acceptance of information technology: toward a unified view. MIS Q.

[24] Ajzen I (1991). The theory of planned behavior. Organ Behav Hum Decis Process.

[25] Hu PJ-H, editor. Evaluating telemedicine systems success: a revised model. System Sciences, 2003. Proceedings of the 36th Annual Hawaii International Conference on; 2003: IEEE.

[26] Sun N, Rau PL (2015). The acceptance of personal health devices among patients with chronic conditions. Int J Med Inform.

[27] Brunelli A, Refai M, Xiume F, Salati M, Marasco R, Sciarra V (2008). Oxygen desaturation during maximal stair-climbing test and postoperative complications after major lung resections. Eur J Cardiothorac Surg.

[28] Brunelli A, Al Refai M, Monteverde M, Borri A, Salati M, Fianchini A (2003). Predictors of exercise oxygen desaturation following major lung resection. Eur J Cardiothorac Surg.

[29] CBS. ICT, kennis en economie 2016. In: Zaken MvE, editor. Den Haag: CBS; 2016.

[30] Courneya KS, Karvinen KH, Vallance JKH, Feuerstein M (2007). Exercise motivation and behavior change handbook of cancer survivorship.

[31] Pinto BM, Ciccolo JT (2011). Physical activity motivation and cancer survivorship. Recent Results Cancer Res.

[32] Achterkamp R**,** Dekker-Van Weering MG**,** Evering RM**,** Tabak M**,** Timmerman JG**,** Hermens HJ et al. Strategies to improve effectiveness of physical activity coaching systems: Development of personas for providing tailored feedback. Health Inform J. 2016**.** 10.1177/1460458216653242 27354396

[33] Op den Akker H, Cabrita M, Op den Akker R, Jones VM, Hermens HJ (2015). Tailored motivational message generation: a model and practical framework for real-time physical activity coaching. J Biomed Inform.

[34] Wolvers M, Bruggeman-Everts FZ, Van der Lee ML, Van de Schoot R, Vollenbroek-Hutten MM (2015). Effectiveness, mediators, and effect predictors of internet interventions for chronic cancer-related fatigue: the design and an analysis plan of a 3-armed randomized controlled trial. JMIR Res Protoc.

[35] Op den Akker H, Jones VM, Hermens HJ (2014). Tailoring real-time physical activity coaching systems: a literature survey and model. User Model User-Adap Inter.

[36] Broens TH, Huis in’t Veld RM, Vollenbroek-Hutten MM, Hermens HJ, van Halteren AT, Nieuwenhuis LJ (2007). Determinants of successful telemedicine implementations: a literature study. J Telemed Telecare.

[37] van Dyk L (2014). A review of telehealth service implementation frameworks. Int J Environ Res Public Health.

[38] Jansen-Kosterink SM. The added value of telemedicine services for physical rehabilitation. Enschede: University of Twente; 2014**.**

[39] Heart T, Kalderon E (2013). Older adults: are they ready to adopt health-related ICT?. Int J Med Inform.

[40] Krijgsman J, Peeters J, Waverijn G, Van Lettow B, Van der Hoek L, De Jong J et al. ‘Omdat ik het belangrijk vind om goed voor mezelf te zorgen’- rapportage eHealth-doelstellingen 2016. Den Haag & Utrecht: Nictiz & Nivel2016.

[41] Bosman R, Bruyckere SD. Dutch Smartphone User - 2016 Q1: Telecompaper 2016.

