# Supplementary material for: Ambulant monitoring and web-accessible home-based exercise program during outpatient follow-up for resected lung cancer survivors: actual use and feasibility in clinical practice
Source: J Cancer Surviv. 2017 Apr 10;11(6):720–31. doi: 10.1007/s11764-017-0611-6 (PMC5671546; doi:10.1007/s11764-017-0611-6)
Supplement: Supplementary file 1 — (DOCX 21 kb) [file 11764_2017_611_MOESM1_ESM.docx]

Sample characteristics

|  | | **Stage 1 (n=10)** | **Stage 2 (n=12)** |
| --- | --- | --- | --- |
| Age, *Median (IQR)* | | 56.6 (52.8-62.8) | 59.5 (54.5-66.0) |
| Sex, *n (%)* | |  |  |
|  | Female | 6 (60%) | 4 (33%) |
| BMI, *Median (IQR)* | | 26.6 (22.8-29.3) | 24.5 (21.3-27.1) |
| Marital status, *n* | |  |  |
|  | Relationship, living together | 7 | 11 |
|  | Single | 1 | -- |
|  | Divorced | 2 | -- |
|  | Widow | -- | 1 |
| Employment status, *n* | | | |
|  | Full-time job | 4 | 1 |
|  | Part-time job | 1 | 3 |
|  | Retired | 2 | 3 |
|  | Unemployed | 3 | 4 |
|  | Voluntary work | -- | 1 |
| Comorbidities, *n* | | 7 | 10 |
| Smoking (yes/quit/never), *n* | | 2 / 5 / 3 | 3 / 8 / 1 |
| Pack years, *Median (IQR)* | | 30.0 (0.0-36.3) | 27.0 (8.0-41.0) |
| Lung function, *Median (IQR)* | |  |  |
|  | FEV_1_ (L) | 3.0 (2.3-3.6) | 2.8 (2.4-3.2) |
|  | % predicted FEV_1_^a^ | 98 (82-105) | 93 (73-98) |
|  | FVC (L) | 4.1 (3.1-5.2) | 4.1 (3.5-4.8) |
|  | % predicted FVC^a^ | 111 (103-119) | 105 (95-112) |
|  | % predicted DLCO^a^ | 87 (71-96) | 67 (62-81) |
| Surgery extent, *n* | |  |  |
|  | Lobectomy | 10 | 8 |
|  | Pneumectomy | -- | 2 |
| Neoadjuvant, *n* | | 1 | 2 |
| Adjuvant, *n* | | 3 | 1 |
| ^a^ percentage of predicted values (from medical record); IQR = interquartile range; BMI = body mass index; FEV_1_ = forced expiratory volume in 1 (first) second; FVC = forced vital capacity ; DLCO = diffusion capacity of the lung for carbon monoxide. | | | |
