# Supplementary material for: Ambulant monitoring and web-accessible home-based exercise program during outpatient follow-up for resected lung cancer survivors: actual use and feasibility in clinical practice
Source: J Cancer Surviv. 2017 Apr 10;11(6):720–31. doi: 10.1007/s11764-017-0611-6 (PMC5671546; doi:10.1007/s11764-017-0611-6)
Supplement: Supplementary file 2 — (DOCX 22 kb) [file 11764_2017_611_MOESM2_ESM.docx]

Critical issues elicitated during stage 1 study

| **Symptom and Physical Activity Monitoring (S&PAM)** | | |
| --- | --- | --- |
|  | **Reported problem** | **Changed for stage 2 study** |
| **Content & Information** | *“Current classification of pain medication is ambiguous and cannot be used in clinical care.”* | Patient reported pain medication automatically classified on portal in: paracetamol, NSAIDs, mild opioid, strong opioid, co-analgetics. |
|  | *“Mood is an important indicator for recovery following lung resection, but is not included in the measurement protocol.”* | Addition of self-reported mood to smartphone monitoring protocol using a subsample of six questions selected from the Multidimensional Mood Questionnaire (MDMQ) to measure valence, calmness, energetic arousal [1, 2]. |
|  | Unclear when physiological data (heart rate or oxygen saturation) is actual missing *or* unreliable due to low quality data. | If data from the zephyr or nonin was missing or was low quality for > 1 minute, this was explicitly stated in the graph on the web portal. |
|  | Text on the portal caused ambiguity about interpretation of the results. | Text was changed and for each displayed result/graph an explanation was provided on the portal. |
| **Navigation & structure** | One of the physicians had to log in for access to the symptom monitoring portal | Accessibility of the symptom monitoring data from the EPR without additional log in was assured. |
| **Design & presentation** | *“Current visualization of pain medication use does not correspond with the graph with pain scores, which makes it difficult to interpret the results quickly.”* | Reports of pain medication were added to/integrated with the graph that visualized pain scores. |
|  | *“Colours of trendlines in graphs do not correspond with the colours indicated in the legend.”* | Colours of the trendlines were adapted. |
|  | Unrealistic value for weight was entered on the smartphone. | For each variable, including weight, unrealistic values were defined, and were rejected by the system. |

| **Web-accessible home-based exercise program (WEP)** | | |
| --- | --- | --- |
|  | **Reported problem** | **Changed for stage 2 study** |
| **Content & Information** | The therapist can only select exercises from an existing database; it is not possible to add an exercise to this database for a specific patient. | Blank exercises added for which therapists can define own text, instructions, and pictures. |
|  | Due to limited contact between therapist and patients, timely adaptation of the exercise program is easily forgotten. | Therapists will receive an automatically generated reminder by email when no exercise program is available the upcoming week. |
| **Navigation & structure** | None | -- |
| **Design & presentation** | Patients cannot perform the exercise session on a different day than prescribed by the therapist, which might hamper adherence. | *Not solved for stage 2 due to time limitation.* Taken as re-design input for next releases. |
|  | Exercise progression is difficult to interpret from the current progression overview, since it summarizes weekly performance. | Summary of performance/progression can be set individually: therapist can select if a summary is needed per day or per week. |
|  | Notifications of new messages remain active/visible on the portal, while all messages have been read by the therapist. This causes confusion whether or not all new messages have been read. | As soon as a therapist clicks on a new message, its notification will disappear from the home screen. |

**References**

1. Kanning M. Using Objective, Real-Time Measures to Investigate the Effect of Actual Physical Activity on Affective States in Everyday Life Differentiating the Contexts of Working and Leisure Time in a Sample with Students. Front Psychol. 2012;3:602.

2. Wilhelm P, Schoebi D. Assessing mood in daily life - Structural validity, sensitivity to change, and reliability of a short-scale to measure three basic dimensions of mood. Eur J Psychol Assess. 2007;23(4):258-67.
