# Supplementary material for: Ambulant monitoring and web-accessible home-based exercise program during outpatient follow-up for resected lung cancer survivors: actual use and feasibility in clinical practice
Source: J Cancer Surviv. 2017 Apr 10;11(6):720–31. doi: 10.1007/s11764-017-0611-6 (PMC5671546; doi:10.1007/s11764-017-0611-6)
Supplement: Supplementary file 3 — (DOCX 57 kb) [file 11764_2017_611_MOESM3_ESM.docx]

**UTAUT component scores for the S&PAM and WEP modules prior to intervention (t0 •, n=12) and post-intervention (t3**
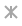
**, n=8).** A patient data point is represented by a dot/cross. Data points that overlap each other are visualized with larger dots/cross; the more overlapping points the larger the dot/cross. The median (horizontal lines) and IQR (error bars) are given. Components: EE=effort expectancy; PE=performance expectancy; ATT=attitude; SI=social influence; PSE=perceived self-efficacy; BI=behavioral intention to use; Satisf=satisfaction.

EE

PE

ATT

SI

PSE

BI

Satisf

EE

PE

ATT

SI

PSE

BI

Satisf
